# Allocation of Secondary Metabolites, Photosynthetic Capacity, and Antioxidant Activity of Kacip Fatimah (*Labisia pumila* Benth) in Response to CO_2_ and Light Intensity

**DOI:** 10.1155/2014/360290

**Published:** 2014-02-10

**Authors:** Mohd Hafiz Ibrahim, Hawa Z. E. Jaafar, Ehsan Karimi, Ali Ghasemzadeh

**Affiliations:** ^1^Department of Biology, Faculty of Science, Universiti Putra Malaysia, 43400 Serdang, Selangor, Malaysia; ^2^Department of Crop Science, Faculty of Agriculture, Universiti Putra Malaysia, 43400 Serdang, Selangor, Malaysia

## Abstract

A split plot 3 by 4 experiment was designed to investigate and distinguish the relationships among production of secondary metabolites, soluble sugar, phenylalanine ammonia lyase (PAL; EC 4.3.1.5) activity, leaf gas exchange, chlorophyll content, antioxidant activity (DPPH), and lipid peroxidation under three levels of CO_2_ (400, 800, and 1200 **μ**mol/mol) and four levels of light intensity (225, 500, 625, and 900 **μ**mol/m^2^/s) over 15 weeks in *Labisia pumila*. The production of plant secondary metabolites, sugar, chlorophyll content, antioxidant activity, and malondialdehyde content was influenced by the interactions between CO_2_ and irradiance. The highest accumulation of secondary metabolites, sugar, maliondialdehyde, and DPPH activity was observed under CO_2_ at 1200 **μ**mol/mol + light intensity at 225 **μ**mol/m^2^/s. Meanwhile, at 400 **μ**mol/mol CO_2_ + 900 **μ**mol/m^2^/s light intensity the production of chlorophyll and maliondialdehyde content was the highest. As CO_2_ levels increased from 400 to 1200 **μ**mol/mol the photosynthesis, stomatal conductance, *f*_*v*_/*f*_*m*_ (maximum efficiency of photosystem II), and PAL activity were enhanced. The production of secondary metabolites displayed a significant negative relationship with maliondialdehyde indicating lowered oxidative stress under high CO_2_ and low irradiance improved the production of plant secondary metabolites that simultaneously enhanced the antioxidant activity (DPPH), thus improving the medicinal value of *Labisia pumila* under this condition.

## 1. Introduction

*Labisia pumila*, locally known as Kacip Fatimah, is a herbaceous plant belonging to the family Myrsinaceae. It is widely scattered in the lowland and hilly rain forests of Malaysia, Thailand, Indochina, The Philippines, and New Guinea [1–3]. Three varieties of *L. pumila* are identified in Malaysia, classified as *L. pumila* var. alata, *L. pumila* var. pumila, and *L. pumila* var. lanceolata [2]. It is usually used as a tonic drink among females, and its indication for women's health may be related to its phytoestrogen effects and having similar chemical structure to estrogen [4]. The varieties most universally utilized by the traditional healers are *L. pumila* var. alata and *L. pumila* var. pumila. Kacip Fatimah has been generally used by generations of Malay women by boiling it in water. It is believed that the decoction drink can induce and facilitate childbirth, as well as being a postpartum medication to help contract the birth channel, to tone the abdominal muscles, and to regain body strength [5, 6]. The other uses of this herb include treatments for dysentery, dysmenorrheal, flatulence, and gonorrhea [7–10].

Recently, it was found that *Labisia pumila* extract contains flavanoids, phenolics, and various bioactive volatile compounds [11]. These compounds include demethylbelamcandaquinone B, fatimahol, dexyloprimulanin, epoxyoleanane glycosides, alkenated phenolics, cerebroside, glycerogalactolipids, and lipids [12]. These compounds have been identified as natural antioxidants that reduce oxidative damage to the human body [13–15]. The concentration of plant secondary metabolites was found to be influenced by environmental conditions such as light intensity, CO_2_ levels, temperature, fertilization, and biotic and abiotic factors which can change the concentration of these active constituents [16, 17]. Changes in the accumulation pattern of secondary metabolites due to environmental factors are, however, the result of changes in both the rate of synthesis and the rate of catabolism [18, 19]. The activity of L-phenylalanine ammonia lyase (PAL; EC 4.3.1.5) determines the extent to which phenylalanine is withdrawn from primary metabolism and enters the general phenylpropanoid pathway [17]. Lately, it was found that the enrichment of *L. pumila* with high levels of CO_2_ increased the secondary metabolite production (phenolics and flavonoids) of this plant [20–25]. Similar results were observed in ginger (*Zingiber officianale*) [24].

Atmospheric CO_2_ concentration has increased by about a third over preindustrial levels and is continuing to increase at around 0.4% per year. By the year 2100, the atmospheric partial pressure of CO_2_ is projected to be 700 parts per million (ppm) or higher [26]. Increased CO_2_ concentration in the air promotes growth and accelerates physiologic processes in young trees because it causes an increase in internal carbon availability and changes partitioning of assimilates among plant parts (roots, stems, and leaves) and primary and secondary metabolites [27]. Plant responses to CO_2_ will likely be influenced by other environmental factors [28], including nutrient and water availability [29, 30] and climate change [31]. Irradiance also directly affects plant growth and phytochemistry [31] and is expected to mediate effects of elevated CO_2_ on plants. Both irradiance and CO_2_ are required resources for photosynthesis and their levels will interactively affect primary and secondary plant metabolism [31]. However, studies on interactive effects between irradiance and CO_2_ levels on phytochemistry of herbal plants were limited. Recently, researchers have reported that environmental factors such as irradiance and CO_2_ concentration can significantly alter secondary metabolite synthesis and production in plants [32–36]. The synthesis of medicinal components in the plant is affected by CO_2_ and light intensity with changes observed in plant morphology and physiological characteristics [37, 38]. Both factors were known to adjust not only plant growth and development but also the biosynthesis of primary and secondary metabolites [39].

Two theories that best describe plant phyto-chemical responses to elevated CO_2_ and different irradiance have received much attention. The carbon/nutrient balance (CNB) theory [40, 41] proposes that allocation to storage and secondary metabolites is determined by the balance between the availability of carbon and nutrients, such that carbon in excess of growth demands is allocated to carbon-based secondary metabolites or storage compounds. The growth-differentiation balance (GDB) theory [42] is premised upon general patterns for relative responses of net assimilation and growth to resource availability (e.g., water, nutrients, and light), such that conditions limiting growth more than assimilation allow resources to be available for differentiation processes such as secondary metabolism.

There have been several studies that investigated the effects of elevated CO_2_ on plant primary and secondary metabolites [25, 43–45], but only a few covered the responses of secondary metabolites under increasing CO_2_ and irradiance. Although secondary metabolites constitute a significant sink for assimilated carbon, to date the picture is unclear as to how these compounds respond to different levels of CO_2_ and irradiance. However, the interaction of CO_2_ enrichment with different irradiance levels is expected to enhance the medicinal value of this plant. Hence, the objective of this study was to examine the effects of different CO_2_ and irradiance levels on the secondary metabolites (total flavonoids, phenolics, anthocyanins, and ascorbic acid), soluble sugars, leaf gas exchange characteristics, phenylalanine ammonia lyase (PAL) activity, lipid peroxidation, and antioxidant activity (DPPH) of *L. pumila* seedlings. The study also aims to investigate at which CO_2_ and irradiance levels the production of secondary metabolites was enhanced. The relationships between all these parameters were also investigated.

## 2. Materials and Methods

### 2.1. Experimental Location, Plant Materials, and Treatments

The experiment was carried out in the glasshouse complex at Field 2, Faculty of Agriculture, Universiti Putra Malaysia (longitude 101° 44′ N and latitude 2° 58′ S, 68 m above sea level), with a mean atmospheric pressure of 1.013 kPa. Three-month-old *L. pumila* var. alata seedlings were left for a month in the nursery to acclimatize until they were ready for the treatments. CO_2_ enrichment treatments were initiated when the seedlings reached four months of age, and when plants were exposed to 400, 800, or 1200 *μ*mol/mol CO_2_. *Labisia pumila* plants were grown under four levels of shade (0, 20, 40, or 60% shade) using black net. The average light intensity passing through each of the 0, 20, 40, and 60% shade treatments was 900, 625, 500, and 225 *μ*mol/m^2^/s, respectively. The photosynthetic photon flux density was measured using LICOR-1412 quantum sensors. When the seedlings had reached 4 months of age, they were fertilized with NPK Blue Special at 15 g per plant. The seedlings were planted in a soilless medium containing coco-peat, burnt paddy husk, and well composted chicken manure in a 5 : 5 : 1 (v/v) ratio in 25 cm diameter polyethylene bags. Day and night temperatures in the glasshouse were maintained at 27–30°C and 18–21°C, respectively. The relative humidity was maintained between 50 and 60%. All seedlings were irrigated using overhead mist irrigation, which was given four times a day or when necessary. Each irrigation session lasted for 7 min. The factorial experiment was arranged in a split plot using a randomized complete block design with the three CO_2_ levels (400, 800, and 1200 *μ*mol/mol) as the main plots and four levels of light intensity (900, 625, 500, and 225 *μ*mol/m^2^/s) as the subplots replicated three times. Each experimental unit consisted of seven seedlings, with a total of 252 seedlings in the experiment. All plants were harvested at the end of 15 weeks of treatments.

### 2.2. Growth Chamber Microclimate and CO_2_ Enrichment

The seedlings were raised in specially constructed growth chambers receiving 12 h photoperiod and average photosynthetic photon flux density of 330 *μ*mol/m^2^/s. Day and night temperatures were recorded at 30 ± 1.0°C and 20 ± 1.5°C, respectively, with a relative humidity of about 70 to 80%. Vapor pressure deficit ranged from 1.11 to 2.32 kPa. Carbon dioxide at 99.8% purity was supplied from a high-pressure CO_2_ cylinder and injected through a pressure regulator into the fully sealed 2 × 3 m growth houses at 2 h daily and applied continuously from 08:00 to 10:00 a.m. The CO_2_ concentrations of the different treatments were measured using Air Sense CO_2_ sensors designated to each chamber during CO_2_ exposition period. Plants were watered three to four times a day at 5 min per session to ensure normal growth of plants using drip irrigation with emitter capacity of 2 L/h [46].

### 2.3. Total Phenolics and Flavonoids Quantification

The extraction and quantification of total phenolics and flavonoids followed the method described by Ibrahim et al. [47]. An amount of ground tissue samples (0.1 g) was extracted with 80% ethanol (10 mL) on an orbital shaker for 120 minutes at 50°C. The mixture was subsequently filtered (Whatman Number 1), and the filtrate was used for the quantification of total phenolics and total flavonoids. Folin-Ciocalteu reagent (diluted 10-fold) was used to determine the total phenolics content of leaf samples. Two hundred *μ*L of the sample extract was mixed with Follin-Ciocalteau reagent (1.5 mL) and allowed to stand at 22°C for 5 minutes before adding NaNO_3_ solution (1.5 mL, 60 g L^−1^). After two hours at 22°C, absorbance was measured at 725 nm. The results are expressed as mg g^−1^ gallic acid equivalent (mg GAE g^−1^ dry sample). For total flavonoids determination, a sample (1 mL) was mixed with NaNO_3_ (0.3 mL) in a test tube covered with aluminium foil and left for 5 minutes. Then 10% AlCl_3_ (0.3 mL) was added followed by addition of 1 M NaOH (2 mL). The absorbance was measured at 510 nm using a spectrophotometer with rutin as a standard (results expressed as mg/g rutin dry sample).

### 2.4. Anthocyanin Content

Anthocyanin content was determined according to Bharti and Khurana [48]. Fresh leaves (1 g) were added in 10 mL acidic methanol (1% v/v HCl) and incubated overnight. Anthocyanin was partitioned from chlorophyll with 10 mL chloroform, followed by addition of 9 mL of double deionised water. The test tubes containing the samples were shaken gently and the mixture was allowed to settle. The absorbance was read at 505 nm. Petunidin was used as a standard. Anthocyanin content was recorded as mg/g petunidin fresh weight.

### 2.5. Ascorbic Acid Content

The ascorbic acid content was measured using the modified method of Davies and Masten [49]. Fresh leaf samples (1 g) were extracted in 1% of phosphate-citrate buffer (pH 3.5) using a chilled pestle and mortar. The homogenate was filtered. The filtrate was added to 1 mL of 1.7 mM 2,6-dichloroindophenol (2,6-DCPIP) in a 3 mL cuvette. The absorbance at 520 nm was read within 10 min of mixing the reagents. The extraction buffer was used as a blank. L-Ascorbic acid was used as a standard. Ascorbic acid was recorded as mg/g L-ascorbic acid fresh leaves.

### 2.6. Total Soluble Sugar Determination

Total soluble sugar was measured spectrophotometrically using the method of Ibrahim and Jaafar [50]. Samples (0.5 g) were placed in 15 mL conical tubes, and distilled water was added to make up the volume to 10 mL. The mixture was then vortexed and later incubated for 10 min. Anthrone reagent was prepared by dissolving anthrone (Sigma Aldrich, St Louis, MO, USA, 0.1 g) in 95% sulphuric acid (Fisher Scientific, USA, 50 mL). Sucrose was used as a standard stock solution to prepare the standard curve for the quantification of sucrose in the sample. The mixed sample of ground dry sample and distilled water was centrifuged at a speed of 3400 rpm for 10 min and then filtered to get the supernatant. A sample (4 mL) was mixed with anthrone reagent (8 mL) and then placed in a water bath set at 100°C for 5 min before the sample was measured at an absorbance of 620 nm using a spectrophotometer (model UV160U; Shimadzu Scientific, Kyoto, Japan). The total soluble sugar in the sample was expressed as mg/g sucrose dry sample.

### 2.7. Phenylalanine Ammonia Lyase (PAL) Activity

Phenylalanine ammonia lyase (PAL) activity was measured using the method described by Martinez-Tellez and Lafuente [51]. The enzyme activity was determined spectrophotometrically by measuring the production of trans-cinnamic acid from L-phenylalanine. Enzyme extract (10 *μ*L) was incubated at 40°C with 12.1 mM L-phenylalanine (90 *μ*L, Sigma) prepared in 50 mM Tris-HCl, (pH 8.5). After 15 min of reaction, trans-cinnamic acid yield was estimated by measuring increase in the absorbance at 290 nm. The standard curve was prepared using trans-cinnamic acid as standard (Sigma) and the PAL activity was expressed as nM trans-cinnamic acid/*μ*g protein/h.

### 2.8. Leaf Gas Exchange Measurement

The measurement was obtained using a closed infrared gas analyzer (LICOR 6400 Portable Photosynthesis System; IRGA, Licor Inc. Nebraska, USA). Prior to use, the instrument was warmed for 30 minutes and calibrated with the ZERO IRGA mode. Two steps are required in the calibration process: first the initial zeroing process for the built-in flow meter and second zeroing process for the infrared gas analyzer. The measurements used optimal conditions set at 400 *μ*mol mol^−1^ CO_2_ 30°C cuvette temperature, 60% relative humidity with air flow rate set at 500 cm^3^ min^−1^, and modified cuvette condition of 800 *μ*mol m^−2^ s^−1^ photosynthetically photon flux density (PPFD). The measurements of gas exchange were carried out between 09:00 and 11:00 a.m. using fully expanded young leaves numbered three and four from the plant apex to record net photosynthesis rate (*A*). The operation was automatic and the data were stored in the LI-6400 console and analyzed using “Photosyn Assistant” software (Version 3, Lincoln Inc, USA). Several precautions were taken to avoid errors during measurement. Leaf surfaces were cleaned and dried using tissue paper before being enclosed in the leaf cuvette. The light response curve was produced following procedures described in Ibrahim and Jaafar [6] to generate the apparent quantum yield and dark respiration rate.

### 2.9. Maximum Quantum Efficiency of Photosystem II (*f*_*v*_/*f*_*m*_)

Measurements of chlorophyll fluorescence were recorded on fully expanded second leaves. Leaves were darkened for 15 minutes by attaching light-exclusion clips to the central region of the leaf surface. Chlorophyll fluorescence was measured using a portable chlorophyll fluorescence meter (Handy PEA, Hansatech Instruments Ltd., Kings Lynn, UK). Measurements were recorded for up to 5 seconds [52]. The fluorescence responses were induced by emitting diodes. Measurements of *f*_0_ (initial fluorescence), *f*_*m*_ (maximum fluorescence), and *f*_*v*_ (variable fluorescence) were obtained. The *f*_*v*_ values were derived as the differences between *f*_*m*_ and *f*_0_.

### 2.10. DPPH Radical Scavenging Assay

The DPPH free radical scavenging activity of each sample was determined according to the method described by Joyeux et al. [53]. A solution of 0.1 mM DPPH in methanol was prepared. The initial absorbance of the DPPH in methanol was measured at 515 nm. An aliquot (40 *μ*L) of an extract was added to 3 mL of methanolic DPPH solution. The change in absorbance at 515 nm was measured after 30 min. The antiradical activity (AA) was determined using the following formula:

(1) AA%=100−[(Abs: sample−Abs: empty  sample)]×100Abs: control.

The optic density of the samples, the control, and the empty samples were measured in comparison with methanol. The synthetic antioxidant, BHT (butylhydroxytoluene), and *α*-tocopherol were used as positive controls.

### 2.11. Malondialdehyde (MDA) Determination

Lipid peroxidation of plant parts was estimated by the level of malondialdehyde (MDA) production using the thiobarbituric acid (TBA) method as described by Alexieva et al. [54]. One gram of ground (0.25 mm) plant sample was homogenized with a mortar and pestle in 0.5% trichloracetic acid (TCA, 1 mL). The homogenate was centrifuged at 9,000 rpm for 20 min. The supernatant (0.5 mL) was mixed with 20% TCA (2.5 mL) containing 0.5% TBA and heated in a boiling water bath for 30 min and allowed to cool in an ice bath quickly. The supernatant was centrifuged at 9,000 rpm for 10 min, and the resulting supernatant was used for determination of MDA content. Absorbance values at 532 nm were recorded.

### 2.12. Statistical Analysis

Data were analyzed using the analysis of variance procedure in SAS version 17. Means separation test between treatments was performed using Duncan multiple range test. The standard error of differences between means was calculated with the assumption that data were normally distributed and equally replicated [55, 56].

## 3. Results and Discussion

### 3.1. Total Phenolics and Flavonoids

Accumulation of total phenolics and flavonoids in *L. pumila* was influenced by the interaction effect between CO_2_ and irradiance (*P* ≤ 0.01; Table 1). Generally, total phenolics and flavonoids was observed to be the highest at 1,200 *μ*mol/mol CO_2_ + 225 *μ*mol/m^2^/s (3.25 mg gallic acid/g dry weight) and was the lowest at 400 *μ*mol/mol CO_2_ + 900 *μ*mol/m^2^/s (0.92 mg gallic acid/g dry weight). Total flavonoids content followed the same trend as total phenolics where the highest total flavonoids content was observed at 1,200 *μ*mol/mol CO_2_ + 225 *μ*mol/m^2^/s irradiance which registered 2.12 mg rutin/g dry. The lowest weight was recorded at 400 *μ*mol/mol CO_2_ + 900 *μ*mol/m^2^/s irradiance which contained only 0.46 mg rutin/g dry weight. The present results are in contrast with a previous study by McDonald et al. [57] who found that the highest bioactive compound was accumulated under elevated CO_2_ and high light conditions in *Populus tremuloides*, *Betula papyrifera*, and *Acer saccharum*. In the present study, the enrichment with high CO_2_ (1200 *μ*mol/mol) under low irradiance (225 *μ*mol/m^2^/s) was found to increase the production of *L. pumila* secondary metabolites. Enhanced production of secondary metabolites under high levels of CO_2_ is usually reported [47–49]. The present results indicate that enrichment of *L. pumila* with high CO_2_ (1200 *μ*mol/mol) with 225 *μ*mol/m^2^/s irradiance can upregulate the production of total phenolics and flavonoids in the plant. The increased production of secondary metabolites under high CO_2_ and low irradiance is attributed to the enhanced availability of soluble carbohydrates under this condition that upregulates the production of total phenolics and flavonoids [58]. Furthermore, the phenolics and flavonoids contents have high significant positive correlationships with soluble carbohydrates (total phenolics *r* ^2^ = 0.901; *P* ≤ 0.05; total flavonoids *r* ^2^ = 0.783; *P* ≤ 0.05; Table 2) which indicates that the up-regulation of secondary metabolites in *L. pumila* under this condition might be contributed by the increase in soluble carbohydrate content. The increase in carbohydrate content might increase the production of *L. pumila* secondary metabolites due to increase in substrates to the shikimic acid pathway [59]. The high total phenolics and flavonoids content under high CO_2_ and low irradiance in the plant has been shown to have anticancer properties and also to have applications for the use as antibiotics, antidiarrhea, antiulcer, and anti-inflammatory agents, as well as in the treatment of diseases such as hypertension, vascular fragility, allergies, and hyperchlolesterolemia [60, 61]. The results imply that CO_2_ enrichment under low irradiance can enhance the medicinal properties of this herb.

### 3.2. Soluble Carbohydrates

The accumulation of soluble carbohydrates in *L. pumila* followed the same trends with total phenolics and flavonoids. The soluble sugar was influenced by the interaction between CO_2_ and irradiance (*P* ≤ 0.05; Table 3). The soluble sugar content was found to be higher as CO_2_ levels increased from 400 to 1200 *μ*mol/mol and as the irradiance decreased from 900 to 225 *μ*mol/m^2^/s with the highest accumulation of soluble sugar observed at 1200 *μ*mol/mol CO_2_ + 225 *μ*mol/m^2^/s irradiance which registered 40.71 mg/g sucrose dry weight, and it was the lowest at 400 *μ*mol/mol CO_2_ + 900 *μ*mol/m^2^/s irradiance that only registered 18.34 mg/g sucrose dry weight. The present results suggest that at high levels of CO_2_ (1200 *μ*mol/mol) under low irradiance (225 *μ*mol/m^2^/s), the production of flavonoids soluble sugars was enhanced. Ghasemzadeh et al. [24] described the accumulation of carbohydrates as a signal of an increase in production of secondary metabolites that enhanced the medicinal quality of plants. The extra carbohydrates accumulated in *L. pumila* seedlings in the present study might be channeled for the production of secondary metabolites (total phenolics and flavonoids) [62–68]. Carbohydrates are the basic compounds required to produce phenolic compounds through the shikimic acid pathway where extra carbohydrates derived from glycolysis and the penthose phosphate pathway are converted into aromatic amino acids [69]. The upregulation in carbon-based secondary metabolites (CBSM) in the present study might be related to the balance between carbohydrate source and sink, as the greater the source-sink ratio, the greater the production of secondary metabolites that might occur [70]. The present findings are in agreement with Guo et al. [71] who found that an increase in sucrose content corresponded with the enhanced production of ascorbic acid, glucosinolates, sulforaphane, anthocyanins, and total phenolics and increased the antioxidative activities in broccoli sprouts. The current results indicate that the exposure of *L. pumila* to high CO_2_ under low irradiance can enhance the health promoting effects of this plant.

### 3.3. Anthocyanin Content

In the present study, anthocyanin content was found to be influenced by the interaction effects between CO_2_ and irradiance levels (*P* ≤ 0.05; Table 3). The accumulation of anthocyanin exhibited a similar pattern with total phenolics, flavonoids, and soluble sugars. In the leaves, the imposition of 225 *μ*mol/m^2^/s irradiance increased anthocyanin production to a maximum at 1200 *μ*mol/mol (0.97 mg/g fresh weight), whilst the lowest anthocyanin content of 0.69 mg/g fresh weight was recorded at ambient CO_2_ levels (400 *μ*mol/mol) under 900 *μ*mol/m^2^/s irradiance. The same patterns that were observed for total phenolics, flavonoid, and soluble sugar accumulation suggest that enhancement of secondary metabolites production in *L. pumila* was optimized under the highest level of CO_2_ (1200 *μ*mol/mol) under low irradiance (225 *μ*mol/m^2^/s). The increase in anthocyanins under high CO_2_ was also observed in *Populus euramericana* [72] and strawberry [73]. Anthocyanins are naturally occurring phenolic compounds responsible for the color of many flowers, fruits, and berries [63]. They are the most important group of water soluble pigments in plants and they have beneficial health effects as antioxidant and anti-inflammatory agents [74, 75]. Tamura and Yamagami [76] reported that anthocyanins possess positive therapeutic value, mainly associated with their antioxidant activity. Improvement in the anthocyanin content in the current study with elevated CO_2_ and low irradiance suggests a possible mechanism for increasing the medicinal quality of *L. pumila*.

### 3.4. Ascorbic Acid Content

Ascorbic acid was found to be influenced by the interaction effects of elevated CO_2_ and irradiance levels (*P* ≤ 0.05; Table 3). The imposition of 1200 *μ*mol/mol CO_2_ + 225 *μ*mol/m^2^/s irradiance significantly increased the ascorbic acid content compared to the other treatments. At the end of 15 weeks, the ascorbic acid content with 800 *μ*mol/mol CO_2_ + 225 *μ*mol/m^2^/s irradiance, 400 *μ*mol/mol CO_2_ + 225 *μ*mol/m^2^/s, 1200 *μ*mol/mol CO_2_ + 500 *μ*mol/m^2^/s, 800 *μ*mol/mol CO_2_ + 500 *μ*mol/m^2^/s, 400 *μ*mol/mol CO_2_ + 500 *μ*mol/m^2^/s, 1200 *μ*mol/mol CO_2_ + 675 *μ*mol/m^2^/s, 800 *μ*mol/mol CO_2_ + 675 *μ*mol/m^2^/s, 400 *μ*mol/mol CO_2_ + 675 *μ*mol/m^2^/s, 1200 *μ*mol/mol CO_2_ + 900 *μ*mol/m^2^/s, 800 *μ*mol/mol CO_2_ + 900 *μ*mol/m^2^/s, and 400 *μ*mol/mol CO_2_ + 900 *μ*mol/m^2^/s was 0.0065, 0.0064, 0.0059, 0.0053, 0.0050, 0.0047, 0.0043, 0.0041, 0.0036, 0.0030, and 0.0024 mg/g, respectively, compared to 0.0071 mg/g with 1200 *μ*mol/mol CO_2_ + 225 *μ*mol/m^2^/s irradiance. There were no previous reports on the combined impact of elevated CO_2_ and irradiance on the accumulation of ascorbic acid. However, previous research had shown that exposure to elevated CO_2_ can enhance the production of ascorbic acid in *Capsicum anuum* [77], tomato [78], and potato [79]. Ascorbic acid had a significant positive correlation with soluble sugar (*r* ^2^ = 0.887; *P* ≤ 0.05) which indicated that the increase in production of ascorbic acid under high CO_2_ and low irradiance can be attributed to the increased carbohydrate content that upregulated the production of this component. The increase in ascorbic acid content in *L. pumila* seedlings is attributed to the high production of TNC under high CO_2_ and low irradiance. TNC (d-glucose) is a precursor for ascorbic biosynthesis in plants, and the more the availability of TNC was, the more the ascorbic acid would be produced in the L-galactose pathway [80, 81].

### 3.5. Phenylalanine Ammonia Lyase (PAL) Activity

**Figure 1:**
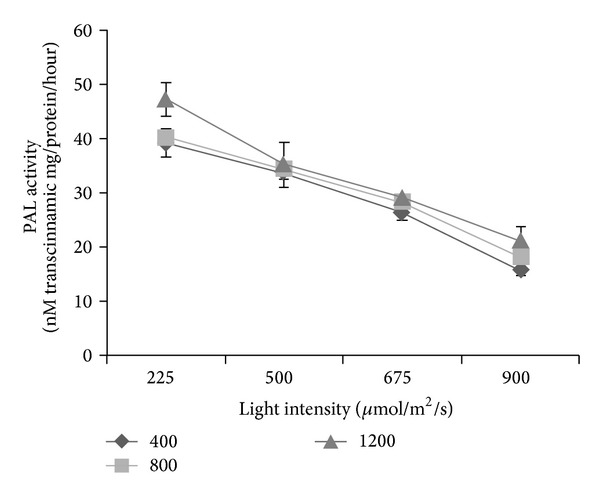
Interaction effects of CO_2_ and irradiance on PAL activity of *Labisia pumila*; *N* = 21. Bars represent standard error of differences between means (SEM).

The phenyl alanine ammonia lyase (PAL) activity was influenced by interaction effects between CO_2_ and irradiance levels (*P* ≤ 0.05; Figure 1). In general it was observed that increase in light intensity reduced the activity of this enzyme in *Labisia pumila*. The highest PAL activity was found to be under 1200 *μ*mol/mol CO_2_ + 225 *μ*mol/m^2^/s irradiance that recorded 47.21 nM transcinnamic mg/protein/hour and the lowest was at 400 *μ*mol/mol CO_2_ + 900 *μ*mol/m^2^/s irradiance, which only registered 15.71 nM transcinnamic mg/protein/hour. The increase in secondary metabolites content in the present study is attributed to the increase in PAL activity under high CO_2_ and low irradiance. Correlation analysis revealed that PAL activity had a significant positive correlation with total phenolics (*r* ^2^ = 0.921; *P* ≤ 0.05) and total flavonoids (*r* ^2^ = 0.895; *P* ≤ 0.05), suggesting an increase in secondary metabolites production with increasing PAL activity. The increase in PAL activity under high CO_2_ and low irradiance might be due to the reduction in nitrogen content under high CO_2_ and low irradiance that enhanced growth and reduced the nitrogen pool in the plant; this restricted the protein production and thus more phenylalanine was available for secondary metabolites production [82, 83].

### 3.6. Total Chlorophyll Content

**Figure 2:**
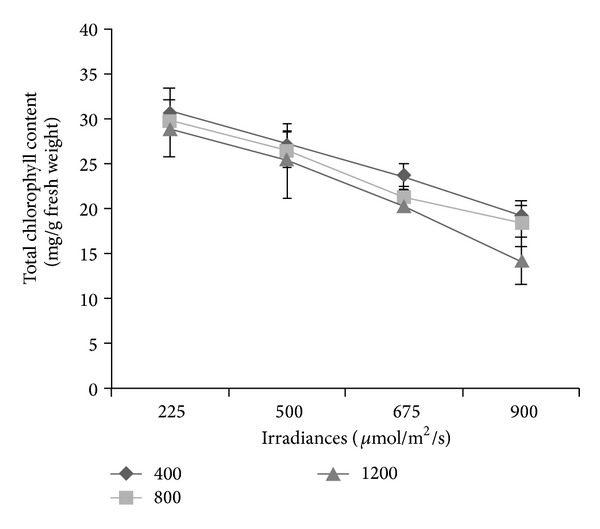
Interaction effects of CO_2_ and irradiance on total chlorophyll content of *Labisia pumila*; *N* = 21. Bars represent standard error of differences between means (SEM).

Similar to PAL activity, the chlorophyll content was also influenced by the interaction between CO_2_ and irradiance levels (*P* ≤ 0.01; Figure 2). The chlorophyll content was found to be the highest at ambient CO_2_ under 225 *μ*mol/m^2^/s irradiance which registered 30.71 mg/g fresh weight and the lowest at 1200 *μ*mol/mol CO_2_ + 900 *μ*mol/m^2^/s irradiance which recorded only 14.21 mg/g fresh weight. The decrease in chlorophyll content with increasing CO_2_ levels has been reported by Porteaus et al. [84] in wheat. The correlations revealed (Table 2) that total chlorophyll were significantly (*P* ≤ 0.05) and negatively related to total phenolics (*r* ^2^ = −0.892), flavonoids (*r* ^2^ = −0.899), anthocyanins (*r* ^2^ = −0.867), and ascorbic acid (*r* ^2^ = −0.846). Competition between secondary metabolites and chlorophyll content fitted well with the prediction of the protein competition model (PCM) and indicated that the secondary metabolites content was controlled by the competition between protein and secondary metabolites biosynthesis pathway and its metabolites regulation [85]. The negative relationship between the secondary metabolites and chlorophyll was a sign of gradual switch of investment from protein to polyphenols production [86]. The present results indicate that under high CO_2_ and low irradiance the production of total chlorophyll content was downregulated in *L. pumila* that also indicated the upregulation on production of secondary metabolites.

### 3.7. Leaf Gas Exchange Properties

**Figure 3:**
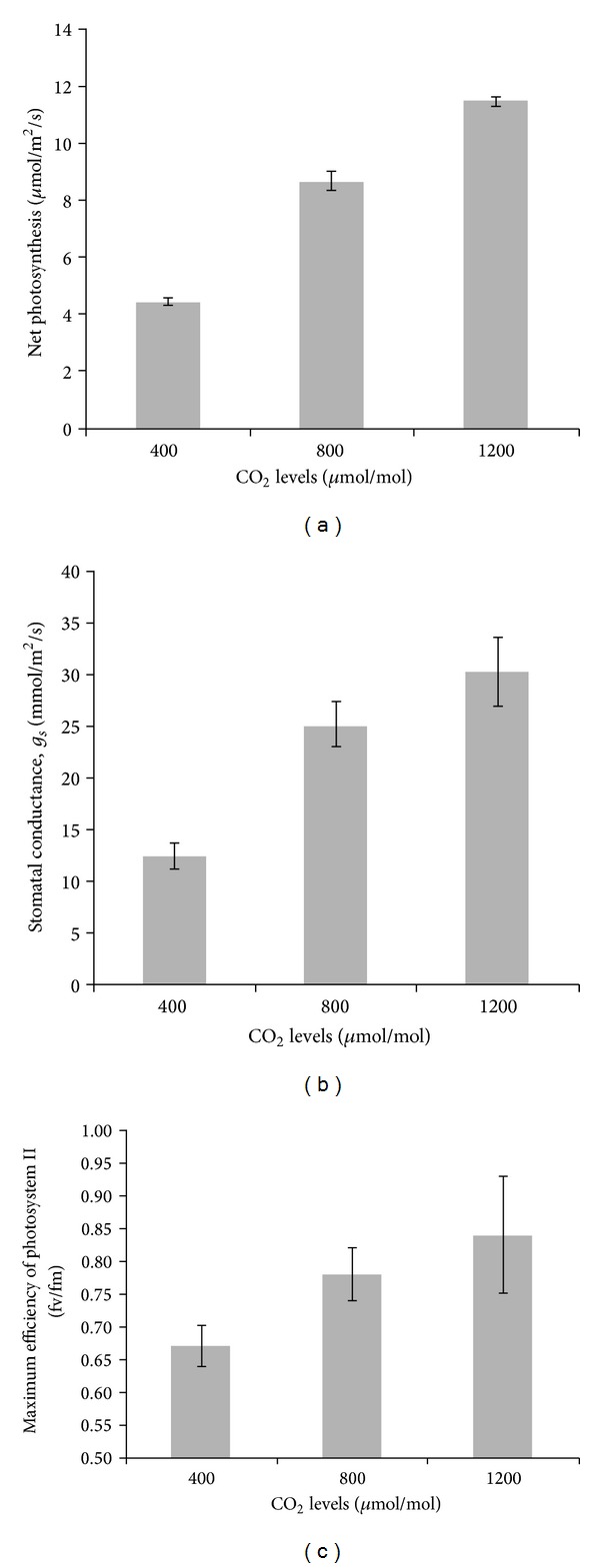
Impact of elevated CO_2_ on (a) net photosynthesis, (b) stomatal conductance, and (c) maximum efficiency of photosystem II (*f*_*v*_/*f*_*m*_) in *Labisia pumila*; *N* = 84. Bars represent standard errors of differences between means (SEM).

The photosynthesis rate, stomatal conductance, and maximum efficiency of photosystem II (*f*_*v*_/*f*_*m*_ ratio) were influenced by CO_2_ levels (*P* ≤ 0.05; Figure 3). No irradiance and interaction effects were observed. Leaf net photosynthesis, stomatal conductance, and *f*_*v*_/*f*_*m*_ rate increased with increasing CO_2_ from 400 to 1200 *μ*mol/mol. The highest net photosynthesis was obtained in *L. pumila* exposed to 1200 *μ*mol/mol CO_2_ (11.45 *μ*mol/m^2^/s), followed by 800 *μ*mol/mol (8.66 *μ*mol/m^2^/s) and 400 *μ*mol/mol (4.44 *μ*mol/m^2^/s; Figure 3(a)). A similar trend was observed for stomatal conductance, where the stomatal conductance for 1200, 800, and 400 *μ*mol/mol CO_2_ was 30.15, 25.12, and 12.40 mmol/m^2^/s, respectively (Figure 3(b)). The *f*_*v*_/*f*_*m*_ ratio was 16 and 25% higher in treatments with 800 and 1200 *μ*mol/mol CO_2_, respectively, compared to the ambient CO_2_ level (Figure 3(c)). These findings exhibit the importance of CO_2_ in further enhancing properties of leaf gas exchange and photosystem II efficiency of *L. pumila* plants. The increase in photosynthesis and photosystem II efficiency in the present work could have stimulated the production of plant secondary metabolites, as suggested by the positive correlation coefficients in Table 2 between photosynthesis and secondary metabolites of total phenolics (*r* ^2^ = 0.987; *P* ≤ 0.05) and flavonoids (*r* ^2^ = 0.786; *P* ≤ 0.05), and also between *f*_*v*_/*f*_*m*_ and total phenolics (*r* ^2^ = 0.887; *P* ≤ 0.05) and flavonoids (*r* ^2^ = 0.846; *P* ≤ 0.05). Thus, an increase in photosynthetic and photochemical efficiency had increased the shikimic acid pathway and enhanced the production of plant secondary metabolites, and this was in turn due to an increase in the concentration of soluble sugars [87, 88]. Some studies had reported that when production of secondary metabolites increased, the photosynthesis would decrease due to feedback control of the secondary metabolites production [89–91]; however, such an effect was not observed in the present study.

### 3.8. DPPH Radical Scavenging Activity

**Figure 4:**
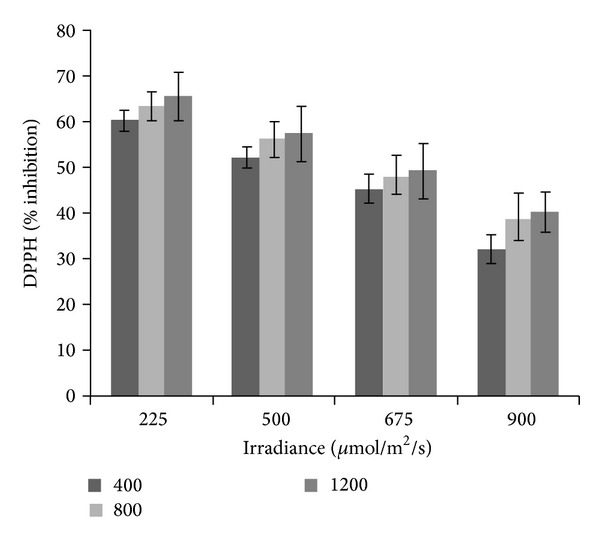
Interaction effect of CO_2_ and irradiance on DPPH scavenging activity of *Labisia pumila*. BHT and *α*-tocopherol were used as controls and valued at 66.41 and 80.41, respectively. *N* = 21. Bars represent standard errors of differences between means (SEM).

DPPH activity was influenced by the interaction between elevated CO_2_ and irradiance levels (*P* ≤ 0.05; Figure 4). The highest DPPH antioxidant activity was recorded with 1200 *μ*mol/mol CO_2_ followed by 800 *μ*mol/mol CO_2_, and it was lowest in the treatment with 400 *μ*mol/mol CO_2_. As the level of irradiance was reduced from 900 to 225 *μ*mol/m^2^/s, the DPPH activity was increased. At 320 *μ*g/mL, the DPPH antioxidant activity recorded the highest inhibition value when exposed to 1200 *μ*mol/mol CO_2_ + 225 *μ*mol/m^2^/s irradiance (65.44%), followed by the 800 *μ*mol/mol + 225 *μ*mol/m^2^/s (63.21%), 400 *μ*mol/mol + 225 *μ*mol/m^2^/s (60.21%), 1200 *μ*mol/mol + 500 *μ*mol/m^2^/s (57.21%), 800 *μ*mol/mol + 500 *μ*mol/m^2^/s (56.23%), 400 *μ*mol/mol + 500 *μ*mol/m^2^/s (52.11%), 1200 *μ*mol/mol + 625 *μ*mol/m^2^/s (49.21%), 800 *μ*mol/mol + 625 *μ*mol/m^2^/s (48.32%), 400 *μ*mol/mol + 625 *μ*mol/m^2^/s (45.27%), 1200 *μ*mol/mol + 900 *μ*mol/m^2^/s (40.21%), 800 *μ*mol/mol + 900 *μ*mol/m^2^/s (39.21%), and the least in the 400 *μ*mol/mol CO_2_ + 900 *μ*mol/m^2^/s irradiance (32.21%) treatment. The results of the current work also showed that the DPPH radical scavenging ability of the plant extracts at 320 *μ*g/mL was less than those of the reference antioxidants butylated hydroxytoluene (BHT, 66.41%) and *α*-tocopherol (80.38%). This study showed that *L. pumila* methanolic extract had good free radical scavenging activity, and, hence, it can be used as a radical scavenging agent acting possibly as a primary antioxidant. The results also imply that exposure to high CO_2_ concentrations under low irradiance could significantly enhance the DPPH radical scavenging activity of the medicinal plant. It must be noted that the DPPH assay principally measures the activity of water-soluble antioxidants [92]. Results of the current work suggest that high CO_2_ supply under low irradiance is advantageous to *L. pumila* in the improvement of antioxidant activity of the water-soluble antioxidants. In our previous study, besides phenolics and flavonoid compounds, other water-soluble antioxidants of the extracts such as ascorbic acid and anthocyanins were suggested to exert additive effects on DPPH radical scavenging activity. This was evident from the current results where DPPH had a significant positive correlationship with total phenolics (*r* ^2^ = 0.923; *P* ≤ 0.05), total flavonoid (*r* ^2^ = 0.887; *P* ≤ 0.05), anthocyanin (*r* ^2^ = 0.887; *P* ≤ 0.05), and ascorbic acid (*r* ^2^ = 0.904; *P* ≤ 0.05). The results imply that the increase in secondary metabolites production under high CO_2_ and low irradiance increases the activity of DPPH.

### 3.9. Lipid Peroxidation Activity

**Figure 5:**
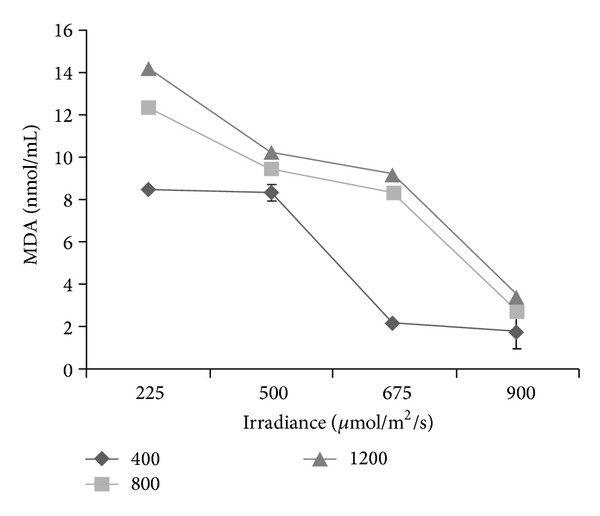
Interaction effects of CO_2_ levels and irradiance on lipid peroxidation DPPH of *Labisia Pumila*; *N* = 21. Bars represent standard errors of differences between means (SEM).

Malondialdehyde (MDA) is a highly reactive three carbon dialdehyde by-product of polyunsaturated fatty acid peroxidation and arachidonic acid metabolism. The production of MDA was influenced by CO_2_ levels imposed onto irradiances (*P* ≤ 0.05; Figure 5). It was observed that as the level of CO_2_ was enhanced from 400 to 1200 *μ*mol/mol CO_2_ the production of MDA was decreased. The MDA content was also found to decrease with increasing irradiance (from 900 to 225 *μ*mol/m^2^/s). This suggests that as CO_2_ levels and irradiance were reduced, the oxidative stress in *L. pumila* cells and tissues was decreased, thus implying occurrence of lipid peroxidation in *L. pumila* under low CO_2_ and high irradiance. Yu et al. [93] working on *P. subcordiformis *had also observed that the reduction in MDA at high concentrations of CO_2_ was due to reduced photorespiration that simultaneously reduced photoreduction of dioxygen. In this case, fewer electrons were transported to dioxygen during photosynthesis and in effect, reducing the potential damage of active oxygen to the membrane system (MDA). The MDA production had established significant negative correlations with total phenolics (*r* ^2^ = 0.876; *P* ≤ 0.05) and total flavonoids (*r* ^2^ = 0.821; *P* ≤ 0.05), indicating that reduction in MDA might be involved in the upregulation of secondary metabolite production under high CO_2_ and low irradiance in *L. pumila* (Table 2). The formation of MDA was considered as a measure of lipid peroxidation that was induced by a high stress level [92]. MDA, a decomposition product of polyunsaturated fatty acid hydroperoxides, has been utilized very often as a suitable biomarker for oxidative stress. Usually the increase in lipid peroxidation is simultaneously accompanied by an increase in hydrogen peroxide levels, but in the present study hydrogen peroxide content was not measured. Hydrogen peroxide may function as a signal for the induction of plant defense systems and this could enhance secondary metabolite production [94]. Hence, the results of the present study suggest that oxidative stress was reduced and was not a prerequisite for secondary metabolite synthesis in *L. pumila* under high CO_2_ and low irradiance.

## 4. Conclusions

The present work has demonstrated that high levels of CO_2_ and low irradiance are able to change the synthesis and allocation of secondary metabolites in *Labisia pumila*. The production of total phenolics, flavonoids, anthocyanins, and ascorbic acid was enhanced as CO_2_ levels increased from 400 to 1200 *μ*mol/mol CO_2_ and irradiance was reduced from 900 to 225 *μ*mol/m^2^/s. The increase in the production of secondary metabolites was followed by an increase in the soluble sugar content. The DPPH antioxidant activity was found to be the highest at 1200 *μ*mol/mol CO_2_ + 225 *μ*mol/m^2^/s irradiance and the lowest under 400 *μ*mol/mol CO_2_ + 900 *μ*mol/m^2^/s irradiance. Elevated CO_2_ significantly improved the leaf gas exchange properties of *Labisia pumila*. The high production of secondary metabolites under high CO_2_ is attributed to increased PAL activity under these conditions. MDA had a significant negative correlation with production of secondary metabolites, indicating that reduction in lipid peroxidation under high CO_2_ and low irradiance in *L. pumila* might improve secondary metabolites production.

## Figures and Tables

**Table 1:** Interaction effects of CO_2_ levels and irradiance on total phenolics and total flavonoids of *Labisia pumila*.

CO_2_ levels (µmol/mol)	Irradiances (*µ*mol/m^2^/s)	Total phenolics (mg gallic acid/g dry weight)	Total flavonoids (mg rutin/g dry weight)
400	225	2.71 ± 0.01^c^	1.54 ± 0.21^c^
	500	2.14 ± 0.03^e^	1.23 ± 0.11^e^
	675	1.64 ± 0.04^g^	0.98 ± 0.91^g^
	900	0.92 ± 0.21^j^	0.46 ± 0.21^j^

800	225	3.01 ± 0.21^b^	1.76 ± 0.03^b^
	500	2.56 ± 0.03^d^	1.42 ± 0.01^d^
	675	1.72 ± 0.01^f^	1.07 ± 0.15^f^
	900	1.36 ± 0.11^i^	0.62 ± 0.02^i^

1200	225	3.25 ± 0.23^a^	2.12 ± 0.04^a^
	500	2.77 ± 0.14^d^	1.50 ± 0.05^de^
	675	1.86 ± 0.24^f^	1.11 ± 0.07^fg^
	900	1.47 ± 0.21^h^	0.86 ± 0.02^h^

All analyses are expressed as means ± standard error of mean (SEM). *N* = 21. Means not sharing a common letter within a column were significantly different at *P* ≤ 0.05.

**Table 2:** Pearson's correlation parameters.

Characteristics	1	2	3	4	5	6	7	8	9	10	11	12
(1) Phenolics	1.000											
(2) Flavonoids	0.899*	1.000										
(3) Sugar	0.901*	0.789*	1.000									
(4) Anthocyanin	0.879*	0.802*	0.567	1.000								
(5) Ascorbic Acid	0.872*	0.823*	0.887*	0.776*	1.000							
(6) PAL activity	0.921*	0.895*	0.665	0.723*	0.712*	1.000						
(7) Chlorophyll	−0.892*	−0.899*	−0.234	−0.567	−0.446	−0.032	1.000					
(8) Photosynthesis	0.987*	0.786*	0.722*	0.668*	0.056	0.456	−0.445	1.000				
(9) S. conductance	0.932*	0.821*	0.678	0.556	0.034	0.332	0.223	0.987*	1.000			
(10) *f*_*v*_/*f*_*m*_	0.887*	0.546	0.801*	0.505	0.021	0.303	0.445	0.887*	0.666*	1.000		
(11) MDA	−0.876*	−0.821*	0.045	0.003	0.042	−0.789*	0.567*	−0.654*	0.324*	0.012	1.000	
(12) DPPH	0.923*	0.887*	0.213	0.887*	0.104	0.901*	−0.445	0.901*	0.023	0.344	−0.455*	1.000

Note. *f*_*v*_/*f*_*m*_: maximum efficiency of photosystem II; PAL: phenyl alanine lyase activity; DPPH: 1,1-diphenyl-2-picryl-hydrazyl (DPPH) assay. *Significant at *P* ≤ 0.05.

**Table 3:** Interaction effects of CO_2_ levels and irradiance on leaf total phenolics and total flavonoids production in *Labisia pumila*.

CO_2_ levels (*µ*mol/mol)	Irradiances (*µ*mol/m^2^/s)	Soluble sugar (mg sucrose/g dry weight)	Anthocyanin (mg petunidin/g dry weight)	Ascorbic acid (mg/g dry weight)
400	225	32.110 ± 2.110^c^	0.860 ± 0.211^b^	0.064 ± 0.001^c^
	500	26.220 ± 0.340^f^	0.701 ± 0.011^c^	0.050 ± 0.003^e^
	675	23.100 ± 0.231^i^	0.610 ± 0.231^e^	0.041 ± 0.004^g^
	900	18.341 ± 1.241^l^	0.371 ± 0.05^h^	0.024 ± 0.003^j^

800	225	35.210 ± 2.152^b^	0.881 ± 0.041^a^	0.065 ± 0.002^b^
	500	28.910 ± 0.452^e^	0.750 ± 0.012^b^	0.053 ± 0.006^d^
	675	24.271 ± 2.441^h^	0.640 ± 0.060^d^	0.043 ± 0.001^f^
	900	19.722 ± 3.212^k^	0.400 ± 0.073^g^	0.030 ± 0.003^i^

1200	225	40.712 ± 2.561^a^	0.920 ± 0.020^a^	0.071 ± 0.002^a^
	500	30.721 ± 2.110^d^	0.790 ± 0.012^b^	0.059 ± 0.001^d^
	675	26.110 ± 0.450^g^	0.671 ± 0.053^d^	0.047 ± 0.004^f^
	900	21.211 ± 3.212^j^	0.480 ± 0.031^f^	0.036 ± 0.003^h^

All results are expressed as means ± standard error of mean (SEM). *N* = 21. Means not sharing a common letter within a column were significantly different at *P* ≤ 0.05.
